# Editorial: Research on nanomaterials in tumor diagnosis and therapy

**DOI:** 10.3389/fbioe.2024.1545581

**Published:** 2025-01-14

**Authors:** Shi Bai, Yue Kang, Shenglong Li, Song Ma, Teruyoshi Sasayama

**Affiliations:** ^1^ Department of Information Engineering, Shenyang University of Technology, Shenyang, China; ^2^ Department of Breast Surgery, Liaoning Cancer Hospital and Institute, Cancer Hospital of Dalian University of Technology, Cancer Hospital of China Medical University, Shenyang, China; ^3^ Liaoning Cancer Hospital, China Medical University, Shenyang, China; ^4^ Institute of Metal Research, Chinese Academy of Sciences (CAS), Shenyang, China; ^5^ Kyushu University, Fukuoka, Japan

**Keywords:** nanotechnology, tumor therapy, drug delivery, magnetic nanomaterials, magnetic particle image

In recent years, nanotechnology has made significant advancements in the medical field, particularly in the diagnosis and treatment of tumors. This Research Topic showcases the latest research achievements in the field of nanomaterials and tumor therapy, focusing on two main areas: the development of anticancer drugs based on organic nanomaterials and the application of magnetic nanomaterials in imaging diagnosis and treatment.

In the realm of organic nanomaterials, Wang et al. have developed ROS-responsive organic nanomaterials that, through laser-triggered mitochondrial targeting, combine photodynamic therapy with chemotherapy to effectively promote mitochondrial apoptosis. Additionally, Takahashi et al. have developed DDS-type NIR absorbers that enhance therapeutic effects in laser photothermal therapy, treating deep-seated lesions while minimizing damage to surrounding healthy tissues. These studies have achieved significant progress in the development of antitumor drugs and the realm of multimodal combination therapies, enhancing therapeutic precision and diminishing drug-related side effects through the use of organic nanomaterials, thus offering valuable insights for their future clinical application.

In the domain of magnetic nanomaterials, research is divided into two subfields: nanomaterials for tumor medical imaging such as magnetic particle imaging (MPI), and tumor-targeted therapy based on magnetic drug delivery systems. Bai et al. have developed a dynamic imaging device for superficial and deep tumors using magnetic nanomaterials as tracers, namely, a handheld MPI device. This technology has been approved by the Chinese Clinical Trial Registration Center to conduct the world’s first intraoperative detection clinical trial for breast cancer based on MPI technology (registration number: ChiCTR2300077785), marking a significant breakthrough in the clinical application of magnetic nanomaterials in MPI technology. Bai et al. have clarified the inducing magnetic field strength and gradient required for various magnetic nanomaterials to achieve pace-controlled induction and synchronized visualization under MPI or MRI through mathematical and physical analysis, as well as biological experiments. This study provides ample references and basis for subsequent drug delivery based on visualizable magnetic nanomaterials targeting the human body. Zhou et al. have developed composite nanomaterials for magnetic targeted drug delivery and sonodynamic combined therapy, using magnetic nanomaterials as carriers. These materials can be controlled by an induced magnetic field to move within the body, achieving precise targeting of tumor areas and delivery of sonosensitizer drugs, enhancing the targeting and efficacy of sonodynamic therapy in ovarian cancer treatment and demonstrating the value of magnetic nanomaterials in multimodal treatment strategies.

Researchers such as Guan et al. (2024) and Miao et al. have provided a review and commentary on the application of nanomaterials in tumor therapy and diagnosis, highlighting the immense potential of nanomaterials in targeted drug delivery, imaging analysis, immunotherapy, gene therapy, and multimodal combined therapies. This provides new strategies and theoretical frameworks for tumor diagnosis and treatment.

As a summary, with the rapid development of nano-processing and characterization technologies, as well as the advances of supporting instruments, nanomaterials have shown significant scientific and clinical potentials for cancer diagnosis and treatment. Based on the paper collation of the Research Topic and the understanding by the editorial team, we strongly recommend further researchers should pay their attention on in-depth consideration of the integration of medicine and engineering technologies. The key to the success of clinical-grade tumor diagnosis and treatment technology using nanomaterials is to understand the whole-cycle of biological and physical characteristics of nanomaterials in physiological states, as well as the limits of supporting instruments at the current stage. We also suggest that researchers may focus on functional nanomaterials which provide additional clinical values, such as the imaging tracer, drug delivery, and hyperthermia ability of magnetic nanoparticles. These new nanoscale capabilities rely more on the physical properties of nanomaterials, which provide innovative complementary support for traditional cancer treatment options based on chemical or biological principles.
